# A City “Collabatory”: Researchers, Commissioners and Community Members Planning Interventions Together

**DOI:** 10.1111/hex.70238

**Published:** 2025-04-01

**Authors:** Neil Small, Rachael H. Moss, Josie Dickerson, Shahid Islam

**Affiliations:** ^1^ Faculty of Health Studies University of Bradford Bradford UK; ^2^ Bradford Institute for Health Research Bradford UK

**Keywords:** Bradford, commissioners, community readiness model, inequalities, interventions

## Abstract

Improving children's health and development in their early years is important, urgent and, cost‐effective. But it's difficult to do. Using an example from Bradford in the UK we argue that integration, innovation and community engagement are key. Long‐term funding also helps. Problems with multiple causes need “whole system” responses. This includes integration of research, commissioning and service delivery. We test innovations, learn about how they are received, modify them and test again. A dynamic research programme starts with innovations that are “science‐based”—things the literature suggests might work—and then evaluates them. Science‐based approaches may translate into being “evidence‐based”. If a community is not ready for an intervention what needs to be put in place to enhance that readiness? We use two examples of using the Community Readiness Model. For obesity interventions in Roma children the model underlines the need to build trust. For interventions targeting social and emotional health, service planners need to explain what they are seeking to do and why it might be valuable. If the community is on‐board, the professionals work together and there is security in broad‐based long‐term funding our “collabatory” approach just might change bringing up children in this city.

## 1 The Context

Bradford in the north of England is the country's 6th largest Metropolitan District. It has considerable ethnic diversity and one of the youngest populations in Europe. There are high levels of deprivation and severe health inequalities. Over the past 10 years multiple interventions seeking to reduce inequalities across the life course have been delivered in the District. We will focus on one of them:

- Better Start Bradford (BSB) implements and evaluates early life interventions focusing on improving the health and development of infants and pre‐school children in three ethnically diverse and deprived areas. It was funded by The National Lottery Community Fund as a part of the “A Better Start” Programme.

There are three key features that underpin the potential of this programme to make a difference:

- It's longevity (2015–2025).
- A funding model that sees research and evaluation funded alongside resources for service innovation and delivery.
- A wide “buy in” to support the aims of the programmes from local government, health, voluntary and community groups.

## 2 Getting Things Done Quickly

There is a paucity of high‐quality research on the effectiveness of interventions that both improve outcomes and promote greater equality in children's health and development. In inner‐city Bradford many children face poor health and entrenched inequalities. Commissioners and practitioners in health, education and social care in the city, as well as researchers, share an urgency to do things *now* to pursue their aim of creating healthier futures for *this* generation of children.

In acknowledging this urgency and the lack of evidence‐based interventions available at the start of their programmes, BSB adopted an approach to interventions that is “science‐based”; that is based on theory as to what might work. The science‐based interventions are then evaluated through the Bradford Institute for Health Research, a research partner with academic, community engagement and data expertise. A key part of evaluation is to take a pragmatic approach, to ‘nudge‐up’ the evidence of interventions step by step—looking at implementation success (feasibility of delivery by services and acceptability in communities) before completing evaluations of effectiveness (improvements in outcomes). Thus, the programme delivery and integrated researcher‐led evaluations create a shift that sees interventions moving from being science‐based to being evidence‐based [1].

Then findings need to be translated speedily into policy and practice. There has been an appetite to take a test and learn approach from commissioners and practitioners. They are seeking to create a system where the services fit the population rather than the population having to fit the services. To achieve this there have been assessments of community readiness and regular monitoring of interventions, with a commitment to adapt recruitment strategies promptly if the project is not reaching the target population [2].

It is a characteristic of health interventions that they emerge when someone (usually a politician or a commissioner) has decided something is a concern of interest, perhaps prompted by public pressure. Decisions are made, oftentimes with input from researchers and practitioners, on how the problem will be addressed and the characteristics of the target population who must be reached. But, waiting in the wings, are the people the intervention is for. Commissioners and service providers know that just because they believe an issue is a priority, this does not mean their concerns will be shared by the community. A commitment, at the ‘pre‐implementation’ stage of any intervention, to assess community readiness has the potential to improve the details of the implementation, its take‐up and its outcomes.

## 3 Identifying Community Readiness

While there are various ways to assess a community's readiness to accept an intervention we focus on one, the Community Readiness Model (CRM). It has five steps: define the community's issue, define the community, identify suitable key respondents (individuals knowledgeable of the chosen community), interview key respondents and score the interviews. An overall community readiness score (and scores from each of these dimensions that contribute to it) acts as a guide for what needs to be put in place before any intervention is attempted [3]. The tool has been widely used internationally and frequently modified to respond to local circumstances.

### 3.1 Examples of How the CRM Has Been Used in Bradford

#### 3.1.1 Obesity in Roma Communities

Roma communities within the UK have a high prevalence of obesity and related health concerns and they have low levels of take‐up of preventative and supportive services. There are structural and systemic barriers to consider, high levels of deprivation and an absence of affordable and accessible food. In addition, there may be wariness about receiving advice from outside their own community that is a legacy of long‐standing marginalisation. Both understanding and enhancing community readiness for any intervention in this context is best achieved by identifying trusted leaders from within the Roma community. They can be a conduit for advice that is tailored to harmonise with the communities' cultural mores and that is sensitive to their structural and historical challenges. Trusted leaders and health professionals can, together, shape education, prevention and treatment. What is decided upon can be offered via intermediaries who are either indigenous to the Roma community or who are closely guided by community members. This is a modification of the CRM that reflects the recognition that trust is the key dimension that needs to be developed before any obesity intervention is attempted [4].

#### 3.1.2 Supporting Mental Health and Wellbeing of Mothers

Perinatal mental ill health is associated with poor child socio‐emotional development and this can lead to a lifelong impact on a child's health and wellbeing. The CRM was used to assess the level of readiness to access services to support mental health for pregnant women and mothers of children aged 0–4 living in a large housing estate. The key findings showed community members were not clear as to what constituted a social or emotional health need or what services were available for them to address such needs. While this finding points to service providers prioritising awareness of: (a) what social and emotional health is and (b) why accessing services may be beneficial it also illustrates a more fundamental challenge in community engagement. The challenges of seeking to look after new babies in the context of social deprivation, limited money and poor housing, may be interpreted as common unhappiness by community members and as mental ill‐health by clinicians. Community engagement requires reciprocal understanding at these points where lifeworld and system world understandings meet [5].

### 3.2 Shortcomings and Strengths of the CRM

The CRM approach has an epistemological challenge, individual change has parallels with change in communities only by analogy. Group processes, including the history of a groups' relationship with the wider society it sits within, and the nature of leadership in that group, create different routes for promoting and complex ways of resisting change. We see these factors in our Roma community example. There is also an inherent problem in defining what constitutes the community to be targeted when one uses the CRM; is it geographic, is it about a shared ethnic identity, is it about shared characteristics, interests and experiences? In our mental health example, it was not clear that being pregnant, or being a new mother in this geographic area, was sufficient to constitute a community identity that could be the target for an intervention focussed on an issue that had such low salience for the targeted group. Both our examples also underline a semantic challenge in negotiating the line between what are best seen as structural barriers that require a collective solution and personal needs that require individual help.

The sorts of intervention planning the CRM sits most comfortably alongside is an iterative approach to innovation and a recognition that this will not work unless the target community can be included in a planning alliance from the outset. It is an approach that changes all parties involved in it—the professionals become more humble and more agile. The dynamic of communities can also change as actions arising from using the CRM encourage individual agency and group solidarities. Being able to say what you think is missing and what would need to be put in place to help you, and then joining with others to look at how best to achieve change, are the sorts of experiences that spill over from the immediate subject of interest to better equip people to intervene in ways that shape the wider determinates of their health and wellbeing.

## 4 Conclusions

If you have commissioners and service providers committed to change course based on levels of community readiness, researchers to provide here‐and‐now information and communities involved in collaborative approaches to shaping interventions, you can begin to ask, ‘can a research study change a city?’ We called such a scenario a “collabatory” in our title. There is a hint of paradox here, it is the severity of Bradford's challenges that help leverage the resources for long‐running interventions and it is the imagination and resolve of its populous, professional and lay, that encourage the whole system responses to entrenched problems that are most likely to succeed.

Doing something about child development in the early years is widely accepted as vital and ‘cost‐effective’. It's one of the core aims of the Labour government's *Plan for Change* announced on 5 December 2024. Our example shows ways of improving outcomes and promoting greater equality by putting the voices of the community into an active alliance with services. It's not an easy or a quick fix, but that's not surprising given that everyone agrees addressing the multi‐factorial causations that are damaging child development in our communities is a challenging aim.

## Author Contributions

**Neil Small:** conceptualization, writing – original draft, writing – review and editing. **Rachael H Moss:** writing – original draft, writing – review and editing, conceptualization. **Josie Dickerson:** writing – original draft, writing – review and editing. **Shahid Islam:** conceptualization, writing – original draft, writing – review and editing.

## Conflicts of Interest

The authors declare no conflicts of interest.

## Patient or Public Contribution

The team included stakeholder and public contribution within each community readiness exploration detailed in this paper.

## Data Availability

The authors have nothing to report.
